# Response of Central Nervous System Biomolecules and Systemic Biomarkers to Aerobic Exercise Following Concussion: A Scoping Review of Human and Animal Research

**DOI:** 10.1089/neur.2024.0062

**Published:** 2024-07-29

**Authors:** Dean M. Cordingley, Izabella Marquez, Serena C. L. Buchwald, Frederick A. Zeiler

**Affiliations:** ^1^ Pan Am Clinic Foundation, Winnipeg, Canada.; ^2^ Applied Health Sciences Program, Faculty of Graduate Studies, University of Manitoba, Winnipeg, Canada.; ^3^ Department of Biosystems Engineering, Price Faculty of Engineering, University of Manitoba, Winnipeg, Canada.; ^4^ Biomedical Engineering, Price Faculty of Engineering, University of Manitoba, Winnipeg, Canada.; ^5^ Department of Surgery, Section of Neurosurgery, Rady Faculty of Health Sciences, University of Manitoba, Winnipeg, Canada.; ^6^ Centre on Aging, University of Manitoba, Winnipeg, Canada.; ^7^ Division of Anaesthesia, Department of Medicine, Addenbrooke’s Hospital, University of Cambridge, Cambridge, United Kingdom.

**Keywords:** exercise testing, exercise treatment, mild traumatic brain injury, physiology

## Abstract

The purpose of this study was to identify the response of biomolecules and biomarkers that are associated with the central nervous system to aerobic exercise in human and pre-clinical models of concussion or mild traumatic brain injury (TBI), and to highlight the knowledge gaps in the literature. A systematic scoping review was conducted following a search of EMBASE, MEDLINE, SCOPUS, BIOSIS, and Cochrane Libraries performed on September 8, 2023 (from data base inception). The scoping review was reported following the Preferred Reporting Items for Systematic Reviews and Meta-Analyses (PRISMA) extension for scoping reviews. Duplicates were removed and article screening was performed using an online systematic review management system. The search resulted in a total of 2,449 articles being identified, with 14 articles meeting the inclusion/exclusion criteria and having their data extracted. One study was conducted in humans, while the remainder of identified studies utilized murine models. The current literature is limited and evaluated many different biomolecules and biomarkers with brain-derived neurotrophic factor being the most researched. Further studies on this topic are needed to better understand the biomarker response to exercise after concussion and mild TBI, especially in the human population.

## Introduction

Concussions persist as a widespread issue, impacting individuals across all age groups resulting in a sustained burden on the health care system. There is a large volume of literature on concussions, however, there is a continued lack of postconcussive treatment options.^1^ Unlike other diseases and injuries, pharmacological treatments are typically not considered when treating concussions,^2^ and treatments targeting the underlying pathophysiology are not plentiful. Exercise therapy following concussion has received substantial attention over the past decade,^1,3–10^ with results showing improvements in recovery time with exercise interventions.^4–7,10–16^ However, the way in which exercise, both an acute bout and chronic exercise therapy, physiologically affects the concussed brain to elicit these beneficial results has not been elucidated.^17^ Autonomic function has been investigated and suggested to contribute to exercise intolerance following a concussion,^18^ however, it is not clear what effect exercise has on the target organ (i.e., the brain) following a concussion. The effects of exercise treatment on cerebrovascular function could also be a mechanism to monitor exercise effects following concussion,^19^ but the current research is not robust enough to draw conclusions.^20–22^ Central nervous system (CNS) biomarkers could be another mechanism to monitor the end-organ impacts of aerobic exercise. Another possible mechanism that could contribute to improved recovery time with aerobic exercise after concussion is the action of exercise-induced secretion of CNS-associated signaling molecules, often referred to as exerkines.^23^

Exercise results in the secretion of numerous exerkines, which, depending on type, communicate in a paracrine, an endocrine, and/or an autocrine manner contributing to the robust physiological response to exercise throughout the body.^23,24^ Existing literature has shown that certain exerkines can act on the CNS.^25^ The release of exercise-induced proteins has been postulated as beneficial in numerous disease conditions such as multiple sclerosis,^26^ dementia,^27^ and in generally promoting cognitive function and brain health.^28^ Exercise elicits changes in growth factors and proinflammatory and anti-inflammatory cytokines, along with other myokines, both systemically and centrally located within the brain.^28^ In addition, biomarkers are often used as a means to monitor the extent of injury and recovery.^29^ The response of these exerkines may contribute to the positive effects of aerobic exercise following mild traumatic brain injury (TBI) and concussion. Therefore, the purpose of this study was to identify the response of various biomolecules and biomarkers to aerobic exercise in human and pre-clinical models of concussion, and to highlight the knowledge gaps in the literature.

## Materials and Methods

The Cochrane Handbook for Systematic Reviews and the scoping review extension for Preferred Reporting Items for Systematic Reviews and Meta-Analyses (PRISMA) were followed to carry out a systematic scoping review.^30^ The protocol for the systematically conducted scoping review was not registered.

### Research question

The research question asked was: “What is the response of CNS biomolecules and systemic biomarkers to aerobic exercise after a concussion/mild TBI?”

For the purpose of this review, concussion was defined as humans with a Glasgow Coma Scale (GCS) score ≥13 who were diagnosed by a health care professional as having a concussion. If the participant’s GCS was not stated, author judgment was used based on descriptive information on the participants such as symptom severity and any limitations in daily activities. For animal models of mild TBI, we required that studies use a mechanical-based injury model (i.e., controlled cortical impact, fluid percussion injury [FPI], weight drop) instead of a pharmacologically induced TBI, and injury severity was based on author description.

Aerobic exercise was defined as any mode of exercise (i.e., cycle ergometer, treadmill, or running wheel) that was at a submaximal intensity.

Biomolecules and biomarkers for this study were defined as any proteins, peptides, messenger, or micro-RNA, which have been implicated as markers of inflammation, cell growth, cell death, or injury severity in the CNS. For a full list of included search terms see Supplementary Data S1 for a sample of the search string.

### Search strategy

A search of EMBASE, MEDLINE, SCOPUS, BIOSIS, and Cochrane Libraries was conducted on September 8, 2023, for all relevant literature from data base inception. The study search strategy was developed by researchers with previous experience in designing and conducting systematic reviews and expertise on the topic (D.M.C. and F.A.Z.). An online systematic/scoping review management software (https://www.rayyan.ai) was used to remove duplicates and for screening the remaining articles by researchers (D.M.C., I.M., and S.C.L.B.). Reference lists for all articles included in the study were searched for any additional studies. Supplementary Data S1contains a sample of the search strategy used, and Supplementary Data S2includes the PRISMA checklist.

### Eligibility criteria

#### Inclusion criteria

Articles were included if they were published in a peer-reviewed journal. Studies needed to have an aerobic exercise intervention (acute or chronic) following a concussion or mild TBI to be included in this article. Both human and animal studies with participants of any age were included. The participants in human studies must have been diagnosed with a concussion or mild TBI only. In studies utilizing an animal model, only mechanically induced mild TBI (i.e., controlled cortical injury, FPI, weight drop) was included.

#### Exclusion criteria

If an identified article was a conference abstract/article, case report, case series, or literature that was no original research, it was excluded. Studies that did not involve an aerobic exercise intervention, or incorporated aerobic exercise interventions combined with additional interventions, making it difficult to discern the individual treatment effects, were excluded. In addition, studies that did not measure a CNS-implicated biomolecule or biomarker in relation to the exercise, included moderate and/or severe TBI populations, utilized a pharmacological model of TBI, and studies that were non-English were excluded.

### Article selection process

All titles and abstracts were reviewed by D.M.C. and I.M. to determine that in all articles a full-text review will be performed. The full text of potential articles was reviewed by two researchers (D.M.C. and I.M. or S.C.L.B.). If a disagreement occurred, researcher F.A.Z. was included to reach consensus.

### Data charting process

Data extraction for all relevant articles was performed using two templates (see Table 1 and Table 2). Data were extracted for study design, participant characteristics, methodologies, aerobic exercise protocol, all physiological outcome measures, detailed biomolecular and biomarker outcomes measured in association with the aerobic exercise, study results specific to the biomolecules and biomarkers implicated in the CNS, and study conclusions. For any biomolecules or biomarkers identified in only one study, they were excluded from Table 1 and Table 2 and included only in Supplementary Data S3.

**Table 1.** Study Characteristics

Reference	Study design	Sample	Mechanism of injury	Study protocol	Aerobic exercise intervention	Relevant biomarkers evaluated
Hicks et al.^31^	Experimental	Male Sprague-Dawley rats	FPI	3 wks of daily exercise or no exercise	Treadmill exercise started at 5 min on day 1 and increased by 5 min per day until 60 min of exercise was completed each day. Control group was handled for 30–60 sec daily.	Hippocampal BDNF mRNA
Griesbach et al.^32^	Experimental	Male Sprague-Dawley rats	FPI	Animals were caged with or without RW postinjury days 0–6 (acute) or 14–21 (delayed)	Animals were housed with or without a RW between 0–6 days or 14–21 days and performed voluntary exercise.	Protein content of: BDNF Synapsin Phosphosynapsin CREB PhosphoCREB
Griesbach et al.^33^	Experimental	Male Sprague-Dawley rats	FPI	Animals were housed with or without a RW during days 0–6 postinjury. Sacrifice occurred day 7 postinjury.	Animals either had access to a RW during days 0–6 postinjury to perform voluntary exercise, or they did not have access.	Protein content of: BDNF Synapsin I, phosophsynapsin I CREB PhosphoCREB
Griesbach et al.^34^	Experimental	Male Sprague-Dawley rats	FPI	Animals received either a mild or moderate FPI and housed with our without a RW during days 0–6 or 14–20 postinjury. Additionally a group of moderate FPI were housed with or without a RW days 29–36. Sacrifice occurred following the animals last exercise session.	Animals were housed either with or without a RW during postinjury days 0–6, 14–20, or 29–36	Protein content for: BDNF Synapsin Phosphosynapsin CREB PhosphoCREB
Chytrova et al.^35^	Experimental	Male Sprague-Dawley rats	FPI	Animals were housed with or without a RW starting 3 days postinjury. An injection of TrkB-Ig was administered 3 days postinjury. Sacrifice occurred 10 days postinjury	Animals were housed either with or without a RW from days 3–10 postinjury and performed voluntary exercise.	Protein content for: BDNF
Griesbach et al.^36^	Experimental	Male Sprague-Dawley rats	FPI	Animals were injected with TrkB-IgG 14 days postinjury and were either provided a RW or not from days 14–20 postinjury. Sacrifice occurred on day 21 postinjury	Animals were either provided a RW or not for days 14–20 postinjury to perform voluntary wheel running	Protein content for: BDNF Synapsin-I CREB
Szabo et al.^38^	Experimental	Male Sprague-Dawley rats	FPI	Animals were housed with or without a RW from days 0–14 postinjury. Sacrifice occurred on day 14 postinjury	Animals assigned access to a RW performed voluntary wheel running between days 0 and 14 postinjury	Protein content for: Synapsin-1
Kim et al.^37^	Experimental	Male Sprague-Dawley rats	CCI	Animals were randomized to forced exercise or no exercise between days 2–12 postinjury. Sacrifice occurred day 12 postinjury	Exercise consisted of forced treadmill running for 30 min per day for 10 days, starting 2 days after injury. Exercise load progressed from 2 m/min for the first 5 min, to 5 m/min for 5 min, and finally 8 m/min for the last 20 min at 0° incline. The Animals who did not exercise were placed in the treadmill for the same period of time as the exercised animals.	Protein content for: Caspase 3 Bax Bcl-2
Mychasiuk et al.^39^	Experimental	Male and female Sprague-Dawley rats	Lateral Impact	Animals were randomized to begin exercise either the same day as the injury, 3 days, or 7 days postinjury. Sacrifice occurred approximately on postinjury day 17.	Animals were provided a RW on their assigned day (either the same day, 3 days, or 7 days postinjury) and initiated voluntary wheel running	qRT-PCR was used to determine: BDNF
Shin et al.^40^	Experimental	Male Sprague-Dawley rats	CCI	Animals were randomized to receive a bone marrow stromal cell transplant or not 7 days following injury. They were then further randomized to perform exercise or no exercise from day 14–42 postinjury and received injections of BrdU during days 14–18. Sacrifice occurred on day 42.	Animals in the exercise condition ran on a treadmill for 30 min each day. The treadmill speed started at 2 m/min for the initial 5 min, increased to 5 m/min for the following 5 min, and then increased to 8 m/min for the remaining 20 min. Treadmill incline remained at 0° for the duration of exercise	Immunohistochemical staining for: Caspase-3 Protein content for: BDNF TrkB Bax Bcl-2
McGeown et al.^44^	Pretest posttest pilot study	Male (*n* = 4) and female (*n* = 5) adolescents and young adults (age range, 14–21 years) with persistent postconcussion symptoms (≥ 8 wks)	Sustained a concussion during sport/recreation activities (*n* = 8) or motor vehicle collision (*n* = 1) and developed persistent postconcussion symptoms.	Prior to the exercise intervention participants provided a saliva sample, completed a computerized neurocognitive assessment (ImPACT) and completed a Balance Error Scoring System protocol (BESS). Participants completed a 4-wk exercise intervention, completing 3 exercise sessions per wk. After each session participants completed the same assessments as they completed before the intervention.	12 exercise sessions were completed over the course of 4 wks. Each session was ∼1h in duration and consisted of a warm-up, stationary cycling, static balance training, and a cool down. The stationary cycling was performed at an intensity of 20% HR reserve for 25 min for the first wk, 30% HR reserve for 30 min the second wk, 40% HR reserve for 35 min the third wk, and 50% HR reserve for 40 min the final wk. The balance training consisted of performing double leg stance with feet side-by-side, single leg stand, and tandem stance. Balance training progressed from 15–20 min over the 12 sessions and alternated wks from a firm surface and a foam surface.	Salivary-BDNF concentration
Ko et al.^41^	Experimental	Male Sprague-Dawley rats	CCI	The exercise treatment was initiated 3 wks after injury and performed for 8 wks. The radial 8-arm maze test was conducted on day 76 post injury to evaluate spatial learning ability. Sacrifice occurred on day 77 after injury.	The exercise treatment consisted of 30 mins of running every day. Running incline was maintained at 0°, while the running speed was initated at 2 m/min for 5 min, increased to 5 m/min for another 5 min, and then increased to 8 m/min for the final 20 min	Immunohistochemistry for the expression of: Caspase-3 Protein expression for: Bax Bcl-2 BDNF TrkB CREB Phosphorylated-CREB
Bharadwaj et al.^43^	Experimental	Male C57Bl/6J mice	CCI	Animals initiated exercise 3 days postinjury and a portion sacrificed on day 7 for biomarker analysis, while the remaining rodents continued exercise 8 wks	Exercise was initiated 3 days following injury when animals had their RWs unlocked and could perform voluntary running.	qPCR of spinal cord tissue for: BDNF

BDNF, brain-derived neurotrophic factor; mRNA, messenger ribonucleic acid; RW, running wheel; CA1, hippocampal cornu ammonis 1; CA3, hippocampal cornu ammonis 3; FPI, fluid percussion injury; CREB, cAMP response element binding protein; Wk, week; TBI, traumatic brain injury; qPCR, quantitative polymerase chain reaction; CCI, controlled cortical impact; Bax, Bcl-2-associated X Protein; Bcl-2, B cell lymphoma-2; PPCS, persistent postconcussion symptoms; TrkB, tyrosine protein kinase B.

**Table 2.** Study Results and Conclusions

Reference	Relevant results	Conclusions
Hicks et al.^31^	BDNF mRNA hybridization density in the CA1 (*p* < 0.02) and CA3 regions (*p* < 0.05) was higher in the animals who exercised compared with those that did not.	Hippocampal BDNF mRNA expression was elevated in specific regions with exercise.
Griesbach et al.^32^	Compared with FPI animals that were sedentary, hippocampal BDNF protein concentration is decreased if exercise is initiated immediately following FPI (*p* < 0.05), but exercise increases BDNF concentration if delayed until 14 days postinjury (*p* < 0.05). Phosphorylated synapsin I is decreased with early exercise (*p* < 0.0005) but not delayed exercise compared with FPI sedentary animals. Total CREB is decreased with exercise following FPI regardless if the exercise is initiated early (*p* < 0.0005) or late (*p* < 0.05) following FPI. No differences between the FPI sedentary and FPI exercise groups were identified for phosphorylated CREB.	Exercise delayed until 14 days post-FPI may be beneficial to recovery and increase hippocampal BDNF protein content, but early exercise (initiated immediately following FPI) may be detrimental.
Griesbach et al.^33^	Exercise following FPI resulted in lower phosphorylated CREB (*p* < 0.005), synapsin I (*p* < 0.005), and phosphorylated synapsin I (*p* < 0.05), compared with the FPI group, which was sedentary.	Voluntary exercise performed early after FPI may result in decreased intracellular signaling proteins and inhibit plasticity.
Griesbach et al.^34^	Following a mild FPI, acute exercise (days 0–6 post-FPI) did not result in increased BDNF (*p* > 0.05), but delayed exercise (days 14–20 post-FPI) resulted in higher BDNF levels (*p* < 0.005) compared with FPI sedentary animals. Exercise did not result in any difference for total CREB, phosphorylated CREB, total synapsin I, or phosphorylated synapsin I.	The optimal time to exercise initiation to elicit increases in neuroplasticity-related proteins is dependent on the severity of TBI. Following mild FPI, exercise performed 2-wks postinjury elicits an increase in BDNF levels, but exercise at any time did not result in changes in the other biomarkers.
Chytrova et al.^35^	Exercise following FPI normalized hippocampal BDNF levels (*p* < 0.001) compared with FPI alone.	The authors found that BDNF plays a central role in exercise-induced promotion of recovery following FPI. Exercise assists in normalizing molecular markers of plasticity and axonal growth following TBI.
Griesbach et al.^36^	Following FPI, exercise promotes the upregulation of mature BDNF (*p* < 0.05), while BDNF blockade prevented this upregulation (*p* < 0.005). Exercise did not result in a difference in synapsin I levels between the FPI sedentary and FPI exercise conditions. Exercise following FPI increased phosphorylated CREB compared with the FPI sedentary group (*p* < 0.05).	Exercise promotes recovery following TBI and this effect may be dependent on the upregulation of BDNF.
Szabo et al.^38^	FPI alone decreased synapsin I compared with control (*p* < 0.01), but exercise following FPI ameliorated this difference (*p* > 0.05).	Exercise manipulates the molecular mechanisms involved with protein function following TBI. Exercise could benefit synaptic plasticity when introduced post-TBI.
Kim et al.^37^	Exercise moderated the TBI-induced increase in caspase-3 expression (*p* < 0.01). The expression of Bax was suppressed with exercise (*p* < 0.01), while Bcl-2 protein expression was elevated (*p* < 0.01) compared with the TBI-only group.	Exercise following TBI may facilitate recovery by assisting in overcoming neuronal apoptosis.
Mychasiuk et al.^39^	In the prefrontal cortex, BDNF gene expression was only suppressed in female animals following TBI, but exercise was able to improve these levels regardless of when exercise was initiated postinjury. However BDNF gene expression was suppressed in both males and females in the hippocampus, and exercise was unable to restore these levels.	Exercise was generally beneficial for gene expression regardless if it was initiated immediately, 3 days, or 7 days postinjury. The exercise effects on biomarkers may be location and sex specific.
Shin et al.^40^	Following TBI, caspase-3 levels were increased above control, however, exercise was able to decrease the number of caspase-3-positive cells in the dentate gyrus (*p* < 0.05). TBI resulted in decreased BDNF and TrkB expression in the hippocampus, while exercise increased the expression compared with the TBI sedentary animals (all *p* > 0.05). Gene expression of Bcl-2 was decreased in the hippocampus while Bax was increased following TBI. Exercise was able to increase Bcl-2 and decrease Bax compared with the TBI sedentary animals.	Exercise managed to improve gene expression within the hippocampus following TBI-induced alterations.
McGeown et al.^44^	No change was observed in salivary BDNF concentration pre- and postexercise program (*p* = 0.78). A correlation (*p* = 0.04; r^2^ = 0.45) was identified where individuals with a lower initial concentration of BDNF saw larger fold-change increase in BDNF concentration following the exercise intervention.	An exercise treatment including aerobic exercise and balance does not alter salivary BDNF concentrations in individuals with PPCS.
Ko et al.^41^	Exercise decreases the number of caspase-3-positive cells in the dentate gyrus, which are increased following TBI (*p* < 0.05). Bax and Bcl-2 protein expression is decreased and increased, respectively, with exercise following TBI compared with the TBI sedentary animals. This results in a lower Bax-to-Bcl-2 ratio (*p* < 0.05). BDNF, TrkB, and phosphorylated CREB protein expressions were all elevated following TBI, while exercise treatment successfully decreased the overexpression of all three proteins (all *p* < 0.05)	Exercise initiated 3 wks after TBI suppresses activation of the CREB/BDNF/TrkB pathway and suppresses apoptosis of neuronal cells induced by injury.
Bharadwaj et al.^43^	Following TBI, lumbar spinal mRNA levels of BDNF are increased compared with control animals (*p* < 0.0001). However, exercise treatment decreased the mRNA levels of BDNF (*p* < 0.01) compared with the TBI sedentary animals.	Exercise treatment decreased spinal mRNA expression of BDNF compared with TBI sedentary animals.

BDNF, brain-derived neurotrophic factor; mRNA, messenger ribonucleic acid; RW, running wheel; CA1, hippocampal cornu ammonis 1; CA3, hippocampal cornu ammonis 3; FPI, fluid percussion injury; CREB, cAMP response element binding protein; Wk, week; TBI, traumatic brain injury; qPCR, quantitative polymerase chain reaction; CCI, controlled cortical impact; Bax, Bcl-2-associated X protein; Bcl-2, B cell lymphoma-2; PPCS, persistent postconcussion symptoms; TrkB, tyrosine protein kinase B.

### Data items

Extracted data included patient characteristics, study design, exercise intervention, outcome measures, specific methods for biomolecule and biomarker measures, study results, and general conclusions of the study. The patient characteristics that were extracted included sample size, age, biological sex, and any relevant injury information.

### Bias assessment

The purpose of this study was to provide an overview of the current literature investigating the biomolecule and biomarker response to aerobic exercise following mild TBI/concussion, a bias assessment was not conducted.

### Synthesis of results

All data extracted from the studies were described to provide an overview of the available studies and their results; therefore, no formal statistical analysis was performed.

## Results

### Search results and study characteristics

**FIG. 1.**
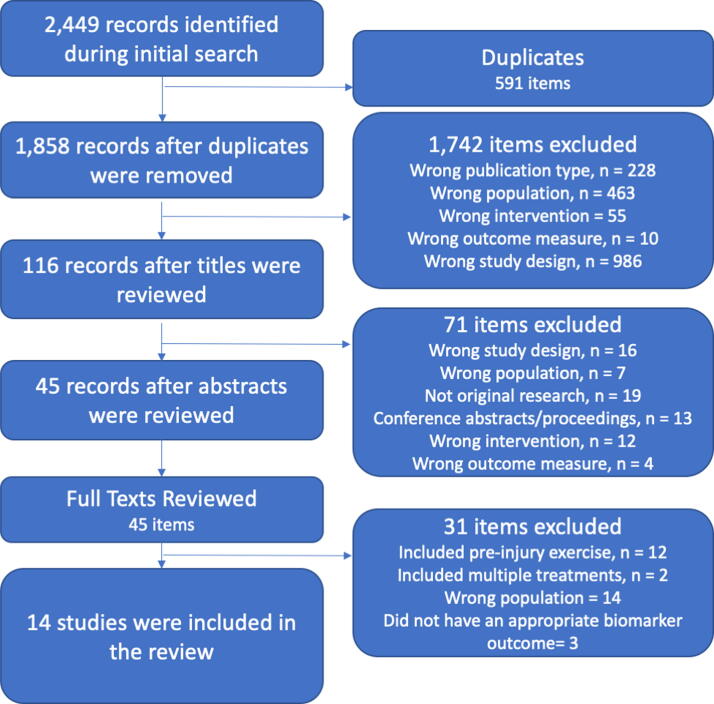
PRISMA flow diagram.

The search strategy resulted in 2,449 articles being identified from all included databases. A total of 591 duplicates were identified and deleted resulting in 1,858 studies for title review. Following screening of article titles, 1,742 were identified as not relevant, leaving 116 articles for abstract screening. The application of the inclusion/exclusion criteria resulted in 45 articles for full-text review. A total of 31 articles were removed following full-text screening because they incorporated preinjury and postinjury exercise as part of their intervention and it was not possible to distinguish between the effects of each (*n* = 12), the brain injury was not a mild TBI or concussion (*n* = 14), they did not evaluate a biomolecule or biomarker implicated with the CNS (*n* = 3), or they included multiple treatments and the effects of exercise alone could not be determined (*n* = 2). The search strategy and inclusion/exclusion criteria resulted in 14 studies being included Fig. 1.

Of the included studies, 13 utilized a murine model,^31–43^ while one used a human-based study design.^44^ The murine models consisted of one that utilized mice,^43^ one that used Wistar rats,^42^ and 11 that used Sprague-Dawley rats.^31–41^ The human study included both males and female participants,^44^ while the majority of murine models only included males,^31–38,40–43^ with the exception of one study that included both males and females.^39^ In addition, the exercise intervention varied between murine studies with some using a treadmill-based intervention to control the exact volume and load of running completed,^31,37,40,41^ while others provided a running wheel for the exercise to be completed.^32–36,38,39,43^ Throughout the studies, there was variation in the biomolecules and biomarkers assessed and the specimens they were evaluated in. For study characteristics, including the biomolecules and biomarkers assessed that were relevant to the purpose of this study, see Table 1, and for biomolecule and biomarker results related to exercise following mild TBI/concussion, see Table 2.

### Biomarker response to aerobic exercise therapy in humans

The search identified one human pilot study that investigated the response of salivary brain-derived neurotrophic factor (BDNF) to an exercise program.^44^ For the study, McGeown et al. recruited 9 participants (age range, 14–21 years old) with persistent postconcussion symptoms (PPCS) (postconcussion symptom scale score, range = 12–78; time since injury, mean = 99.8 ± 79.95 days, range = 51–668 days). Before and after the exercise program, participants provided a saliva sample for BDNF analysis, completed the ImPACT battery of tests, and performed the Balance Error Scoring System test on a force platform. The exercise intervention consisted of 12 one-hour supervised exercise sessions, consisting of both aerobic cycling and balance training, which progressed in difficulty after every 3 sessions. Exercise sessions 1–3 included 25 min of cycling at an intensity of 20% the participants’ heart rate reserve (HRR), and 15-sec balance holds for the double leg stance, single leg stance, and tandem stance on a firm surface with eyes open. Exercise sessions 4–6 included 30 min of cycling at 30% HRR, and 15-sec balance holds on foam with eyes open. Exercise sessions 7–9 included 35 min of cycling at 40% HRR, and 20-sec balance holds on a firm surface with eyes closed. Finally, sessions 10–12 included 40 min of cycling at 50% HRR, and 20-sec balance holds on a foam surface with eyes closed. The exercise treatment did not result in any difference in salivary BDNF concentration. However, the participants pre-exercise treatment salivary BDNF concentration was correlated with the fold-change in concentration found post-treatment (*p* = 0.04, r^2^ = 0.45), where individuals with lower initial salivary BDNF concentrations showed a greater fold-increase in concentration post-treatment compared with individuals who had higher initial BDNF concentrations. McGeown et al.^44^ provide the only evidence of the effects of exercise therapy on biomarkers associated with the CNS following concussion in a human population.

### Biomolecule response to aerobic exercise therapy in animal models

#### BDNF

The most commonly investigated biomarker was BDNF.^31–36,39–41,43^ The identified literature presents a mix of results regarding how exercise changes BDNF levels. A study by Hicks et al.^31^ was the earliest article identified in the search and they found that 3 weeks of treadmill exercise increases hippocampal BDNF messenger ribonucleic acid (mRNA) content following FPI compared with sedentary animals. However, the timing of exercise initiation could be important as it has been found that voluntary wheel running performed for the first 7 days following mild TBI decreases or does not change hippocampal BDNF content compared with being sedentary following mild TBI,^32–34^ but if the exercise is initiated 2 weeks following injury and performed for 7–28 days, the BDNF protein content increases.^32,34,36,40^ Another study that investigated the effects of exercise on BDNF early after mild TBI showed that voluntary exercise initiated 3 days postinjury and continued for 7 days negates the mild TBI-induced decrease in BDNF content and returns it to the level of the control animals (mild TBI and no exercise, BDNF content is 68.1% of the controls vs. mild TBI and exercise, BDNF content is 94.2% of the controls).^35^ Furthermore, delaying exercise initiation to 3 weeks postinjury may result in a much different response. Eight weeks of treadmill exercise following this delayed initiation results in a lower BDNF protein expression in the hippocampus of Sprague-Dawley rats compared with a mild TBI sedentary group of animals who had elevated BDNF protein expression at this time point.^41^

Interestingly, brain region and biological sex may also factor into the observed results in the various studies. A study by Mychasiuk et al.^39^ found that mild TBI resulted in a decrease in hippocampal BDNF gene expression for both male and female Sprague-Dawley rats, with exercise providing no benefit. In addition, BDNF gene expression in the prefrontal cortex of male rats was not changed and exercise provided no changes. However, in the prefrontal cortex of the female rats, mild TBI resulted in a decrease in BDNF gene expression, which was ameliorated with exercise. It is important to note that this study provided running wheel access at 0, 3, or 7 days postinjury and all animals were sacrificed at approximately 17 days postinjury resulting in a different total volume of exercise performed over the treatment period. In addition, biomarker analysis away from the target organ (i.e., the brain) may present differing results than when evaluated locally. BDNF mRNA expression in the lumbar spine of mice was elevated at 7 days after mild TBI, but this increased level was normalized to control levels with 4 days of voluntary wheel running (days 3–7 after mild TBI).^43^

A couple of studies investigated the BDNF receptor tyrosine kinase B (TrkB) content in the hippocampus following exercise in animals with mild TBI.^40,41^ A study by Shin et al.^40^ showed that relative TrkB protein expression is elevated with 28 days of treadmill exercise initiated 2 weeks after mild TBI compared with animals who are sedentary following injury. However, Ko et al.^41^ found contrasting results where relative TrkB protein expression was suppressed with an 8-week treadmill exercise treatment initiated 3 weeks after mild TBI.

#### CREB

Cyclic AMP response element-binding protein (CREB) assists in regulating BDNF gene expression and neurotrophin responses within neurons.^45^ One study found that 7 days of voluntary wheel running initiated acutely following mild TBI results in lower levels of phosphorylated CREB in the parietal cortex and occipital cortex, but no change in total CREB.^33^ However, another study by the same group found that a 7-day voluntary wheel running intervention initiated acutely following mild TBI does not change hippocampal phosphorylated CREB levels, but decreases total CREB in the ipsilateral side of the hippocampus compared with the animals who are sedentary following mild TBI.^32^ In the same study they also showed that 7 days of voluntary wheel running initiated 2 weeks following mild TBI, total CREB protein levels in the ipsilateral hippocampus remain lower compared with the sedentary animals, but there was no difference in phosphorylated CREB.^32^ In contrast to these previous findings, the same group conducted another study and found that following either acute (initiated immediately following mid TBI) or delayed (initiated 14 days following mild TBI) 7-day voluntary wheel running, intervention did not result in any change in total or phosphorylated CREB compared with the sedentary animals.^34^ Conversely to the previously discussed studies which suggested that exercise results in lower CREB protein levels compared with sedentary animals, one study found that 7 days of exercise initiated at 2 weeks after mild TBI results in elevated levels of phosphorylated CREB compared with the animals who were sedentary following mild TBI.^36^

The previous studies all utilized voluntary wheel running as their exercise intervention, but there is one study that used a forced treadmill running protocol.^41^ The exercise intervention was initiated 21 days after mild TBI and performed for 8 weeks. This study found that the 8-week exercise intervention following mild TBI resulted in a decreased ratio of phosphorylated CREB/total CREB protein expression in the hippocampus compared with sedentary animals.^41^

#### Synapsin I

Synapsin I contributes to the regulation of glutamate release from nerve endings,^46^ and was another commonly investigated biomarker identified in this study.^32–34,36,38^ Some research suggests that voluntary wheel running initiated acutely after mild TBI and sustained for 7 days results in a lower protein content of total synapsin I and phosphorylated synapsin I in the parietal cortex and hippocampus compared with sedentary animals following mild TBI,^32,33^ but this finding does not always reach statistical significance.^34^ However, if voluntary wheel running is initiated 2 weeks after mild TBI, there is no difference in hippocampal total or phosphorylated synapsin I compared with the sedentary group.^32,34,36^ It is interesting to note that if voluntary wheel running is initiated acutely after mild TBI but sustained for 14 days, synapsin I protein content is similar to control uninjured animals with exercise, while sedentary animals have decreased synapsin I protein content.^38^ The current research on the effects of exercise on synapsin I following mild TBI and concussion is mixed and requires further elucidation.

#### Biomolecules associated with apoptosis

The most commonly investigated apoptotic protein marker investigated in the identified studies was caspase-3.^37,40,41^ Of these studies, the results consistently demonstrated that exercise treatment following mild TBI decreased the expression of caspase-3 in the hippocampus when compared with animals who were sedentary following mild TBI.^37,40,41^ All studies utilized a treadmill-based exercise treatment, but one study initiated the exercise 2 days following mild TBI,^37^ while the two other studies implemented the exercise treatment either two or three weeks after mild TBI.^40,41^

Two studies investigated the response of Bax (Bcl-2-associated X protein) and Bcl-2 (B cell lymphoma-2) to exercise treatment following mild TBI.^37,41^ A study by Kim et al.^37^ investigated 10 days of treadmill running (30 min per day), which was implemented on day 2 after mild TBI. They saw that exercise treatment decreased the relative Bax protein expression and increased the relative Bcl-2 protein expression compared with animals who were sedentary after mild TBI, resulting in a more favorable Bax/Bcl-2 ratio.^37^ Similarly, Ko et al.^41^ found that 30 min per day of treadmill exercise initiated 3 weeks after mild TBI and performed for 8 weeks resulted in suppressed Bax protein expression, enhanced Bcl-2 expression, and a more favorable Bax/Bcl-2 ratio.

#### Other biomolecules and biomarkers

A myriad of additional biomolecules and biomarkers were assessed in only one study each not making it possible to compare the results from various studies (see Supplementary Data S3 for a full list).

## Discussion

The purpose of this study was to identify the response of CNS-implicated biomolecules and biomarkers to aerobic exercise following concussion, and to highlight the knowledge gaps in the literature. The most significant finding of this systematically conducted scoping review was that only one research study in humans with PPCS has been conducted examining the salivary response of BDNF to exercise therapy.^44^ The remaining studies all utilized murine models and evaluated a large range of biomolecules (26 different biomolecules). The most common biomolecules evaluated were BDNF, TrkB, CREB, synapsin I, caspase-3, Bax, and Bcl-2. This review identified that the available literature used primarily murine models that evaluated varying biomolecules from isolated brain tissue, used different exercise modalities, incorporated different exercise intervention durations, and initiated the exercise interventions at varying time points. Synthesizing this available literature to make clinically meaningful conclusions for the human concussion/mild TBI population is not possible. A clear need for more research in human populations has been identified.

Of the biomolecules and biomarkers investigated in the identified studies, BDNF was the most researched. The function of BDNF in the brain is multifaceted as it is important for neuronal survival and growth, regulating neurotransmitters, and contributes to neuroplasticity.^47^ The sole human pilot study found that BDNF does not change with an exercise intervention in individuals with PPCS.^44^ However, the murine models seem to support that exercise initiated immediately following mild TBI decreases brain BDNF content,^32–34^ while delaying the exercise intervention two weeks seems to increase brain BDNF content.^32,34,36,40^ This finding is interesting because in humans, the initiation of aerobic exercise acutely following concussion has been found to improve time to recovery.^48^ One consideration that was overlooked in the current literature is the possible role that BDNF single-nucleotide polymorphisms (SNPs) may play in patient outcomes following TBI.^49,50^ Future research considering the BDNF SNPs that individuals carry and their outcomes following exercise therapy could establish a more personalized treatment option by identifying those who may, or may not, respond to exercise treatment.

Unlike BDNF, there were much fewer studies that investigated the biomolecules CREB, synapsin I, caspase-3, Bax, and Bcl-2. The biomolecules CREB and synapsin I were each investigated in five studies, but the results were highly variable making it irresponsible to attempt to draw conclusions. The biomolecules associated with apoptosis were investigated in fewer studies, but showed consistent results where exercise treatment decreased markers of apoptosis and promoted an upregulation of antiapoptotic marker Bcl-2. Further research on all of the above biomolecules is needed to assist in generating a complete picture of the physiological response to aerobic exercise in the concussion or mild TBI population.

As previously mentioned in the results, there was a variety of biomolecules that were only evaluated in one of the identified studies. These biomolecules included markers of inflammation, cellular injury, oxidative stress, plasticity, blood–brain barrier integrity, and mitochondrial biogenesis.^33,35,37–40,42,43^ Biomolecules for these various processes are of interest as research groups race to develop diagnostic biomarker panels for concussion and mild TBI.^51^ The literature identified during our review shows a transition to including these types of markers in the exercise therapy-based concussion research, but there remains a paucity of data leaving this area fruitful for future investigation.

### Limitations of the literature

The primary limitation of the current literature is the lack of human data available. The clinical translation of murine TBI models to human is difficult and has historically had limited success.^52,53^ In addition, the included literature had many differences in their exercise intervention, including type of exercise completed (i.e., voluntary wheel running vs. forced treadmill running), the day on which the exercise intervention was initiated following mild TBI, and the duration that the intervention lasted. Also, there were no identified studies that investigated the acute response of biomolecules or biomarkers to a single bout of aerobic exercise following mild TBI. The included studies also used a mix of injury models, each having their own pros and cons, but possibly inducing a slightly different type of concussive/mild TBI injury.^54^ Another limitation of the literature is that only two studies included female participants, one in human participants^44^ and one in animals.^39^ The lack of females does not provide enough information on how biomarkers may respond to aerobic exercise in females with a concussion or mild TBI compared with males.

### Limitations of this review

Studies that directly stated the severity of their TBI model as moderate or severe were excluded from the study. However, many studies did not explicitly state the severity of their TBI model, and if it was not possible to determine upon review of the research, the research was included in the scoping review. In addition, only research published in the English language was included in this study due to the limitations of the authors’ ability to read and understand other languages. Furthermore, drawing conclusions from this review is not possible due to the limited research on each biomolecule and biomarker identified.

### Future directions

Future research to understand the biomarker response to aerobic exercise following a concussion or mild TBI is needed to elucidate underlying mechanisms associated with the therapeutic effects of aerobic exercise in humans.^1,55^ With the ever improving technology to allow for biomarker analysis with smaller volumes of sample, and advancements in neuroimaging to facilitate biomarker identification in living individuals, more human research would be valuable. For humans, systemic biomarkers are typically the most accessible, and therefore, it would be of interest for future research to identify whether systemic biomarker response to aerobic exercise following concussion is related to cerebral physiological changes and patient outcomes. Finally, this review identified a lack of data on females. Sex and gender are both important considerations for concussion research to ensure a complete understanding of patient outcomes for all populations.^56,57^ In addition, to generate a more complete understanding of the impact of exercise on CNS recovery, research should include the following: (1) live time cerebral physiological measures pre-, post-, and long-term in follow-up; (2) serial biomarker measures of both neurotrophic and markers of injury; (3) serial neuroimaging with quantified tractography and connectivity assessments; and (4) serial collection of meaningful patient-reported outcome measures.

## Conclusion

The available literature investigating the response of biomolecules and biomarkers to exercise treatment following a concussion or mild TBI is too limited to draw definitive conclusions on the role they may play in recovery. More research on the physiological response and adaptations, at the level of the brain, in response to aerobic exercise in the concussed population is needed with abundant opportunities for progress. Improving our understanding in this area could possibly assist in the development of more refined exercise treatments following brain injury.

## Abbreviations Used

Bax: Bcl2-associated X Protein
BCL-2: B-cell Lymphoma-2
BDNF: brain derived neurotrophic factor
CNS: central nervous system
CREB: cyclic AMP response element-binding protein
GCS: Glasgow coma scale
HRR: heart rate reserve
ImPACT: Immediate post-concussion assessment and cognitive test
mRNA: messenger ribonucleic acid
PPCS: persistent post-concussion symptoms
PRISMA: preferred reporting items for systematic reviews and meta-analyses
RNA: ribonucleic acid
SNPs: single-nucleotide polymorphisms
TBI: traumatic brain injury
TrkB: tyrosine kinase B
