# Supplementary material for: Response of Central Nervous System Biomolecules and Systemic Biomarkers to Aerobic Exercise Following Concussion: A Scoping Review of Human and Animal Research
Source: Neurotrauma Rep. 2024 Jul 29;5(1):708–20. doi: 10.1089/neur.2024.0062 (PMC11301856; doi:10.1089/neur.2024.0062)
Supplement: Supplementary Data S1 [file neur.2024.0062_supplementalfilea.pdf]

Supplemental File A: Search Terms and Conditions

SCOPUS

(KEY("Mild traumatic brain injury") OR KEY ("Mild TBI") OR KEY ("Brain injury") OR KEY ("Head injury") OR KEY ("Head trauma") OR KEY ("Cerebral trauma") OR KEY ("Brain trauma") OR KEY ("Cerebral Injury") OR KEY ("Concussion") OR KEY ("Closed brain injury") OR KEY ("Acute brain injury") OR KEY ("Brain injuries") OR KEY ("Cerebrovascular trauma") OR KEY ("Mild concussion") OR KEY ("Cerebral Concussion") OR KEY ("Brain Injuries, Traumatic"))

AND

(KEY ("Exercise") OR KEY ("Physical activit*") OR KEY ("Aerobic exercise") OR KEY ("Light intensity exercise") OR KEY ("Moderate intensity exercise") OR KEY ("Exercise training") OR KEY ("Acute exercise") OR KEY ("Leisure Activit*") OR KEY ("Recreation"))

AND

(KEY ("Proteins") OR KEY ("Peptides") OR KEY ("myelin") OR KEY ("neurofilament protein") OR KEY ("ubiquitin") OR KEY ("Glial fibrillary acidic protein") OR KEY ("S100") OR KEY ("micro RNA") OR KEY ("brain derived neurotrophic factor") OR KEY ("BDNF") OR KEY ("Biomarker*") OR KEY ("Marker, Biological") OR KEY ("Biologic Marker") OR KEY ("Marker, Biologic") OR KEY ("Biological Markers") OR KEY ("Biologic Markers") OR KEY ("Markers, Biologic") OR KEY ("Biomarker") OR KEY ("Markers, Biological") OR KEY ("Markers, Immunologic") OR KEY ("Immune Markers") OR KEY ("Markers, Immune") OR KEY ("Marker, Immunologic") OR KEY ("Immunologic Markers") OR KEY ("Immune Marker") OR KEY ("Marker, Immune") OR KEY ("Immunologic Marker") OR KEY ("Serum Markers") OR KEY ("Markers, Serum") OR KEY ("Marker, Serum") OR KEY ("Serum Marker") OR KEY ("Biochemical Marker") OR KEY ("Markers, Biochemical") OR KEY ("Marker, Biochemical") OR KEY ("Biochemical Markers") OR KEY ("Tau") OR KEY ("Proteins, tau") OR KEY ("tau Protein") OR KEY ("Protein, tau") OR KEY ("Chemokine*") OR KEY ("Chemotactic Cytokine") OR KEY ("Cytokine, Chemotactic") OR KEY ("Intercrines") OR KEY ("Chemotactic Cytokines") OR KEY ("Cytokines, Chemotactic") OR KEY ("Intercrine") OR KEY ("Interferon") OR KEY ("Interleukin*") OR KEY ("Leukemia Inhibitory Factor") OR KEY ("Inhibitory Factor, Leukemia") OR KEY ("Differentiation-Stimulating Factor") OR KEY ("Differentiation Stimulating Factor") OR KEY ("D Factor") OR KEY ("Myeloid Differentiation-Stimulating Factor") OR KEY ("Differentiation-Stimulating Factor, Myeloid") OR KEY ("Myeloid Differentiation Stimulating Factor") OR KEY ("Cholinergic Differentiation Factor") OR KEY ("Differentiation Factor, Cholinergic") OR KEY ("Emfilermin") OR KEY ("LIF") OR KEY ("Lymphokines") OR KEY ("Lymphocyte Mediators") OR KEY ("Tumor necrosis factor") OR KEY ("Necrosis Factors, Tumor") OR KEY ("TNF Receptor Ligands") OR KEY ("Receptor Ligands, TNF") OR KEY ("Tumor Necrosis Factor Superfamily Ligands") OR KEY ("Adipokines") OR KEY ("Adipokine") OR KEY ("Adipocytokine") OR KEY ("Adipocytokines") OR KEY ("Inflammation") OR KEY ("Inflammations") OR KEY ("Innate Inflammatory Response") OR KEY ("Inflammatory Response, Innate") OR KEY ("Innate Inflammatory Responses") OR KEY ("Myelin basic protein"))
