# Supplementary material for: Response of Central Nervous System Biomolecules and Systemic Biomarkers to Aerobic Exercise Following Concussion: A Scoping Review of Human and Animal Research
Source: Neurotrauma Rep. 2024 Jul 29;5(1):708–20. doi: 10.1089/neur.2024.0062 (PMC11301856; doi:10.1089/neur.2024.0062)
Supplement: Supplementary Data S3 [file neur.2024.0062_supplementalfilec.pdf]

Supplemental File C: Supplemental Tables

Supplemental Table A: Study characteristics not included in the primary tables.

Reference	Study Design	Sample	Mechanism of Injury	Study Protocol	Aerobic exercise intervention	Relevant biomarker
Griesbach et al [33]	See Table 1.	See Table 1.	See Table 1.	See Table 1.	See Table 1.	CAMKII<br>Phospho-p44/p42<br>MAP kinase I and II<br>PKC
Chytrova et al [35]	See Table 1.	See Table 1.	See Table 1.	See Table 1.	See Table 1.	MAG<br>Nogo-A
Szabo et al [38]	See Table 1.	See Table 1.	See Table 1.	See Table 1.	See Table 1.	Chymotrypsin like activity of proteasome complex<br>Zif 268<br>20S alfa core protein of 26S proteasome complex
Kim et al [37]	See Table 1.	See Table 1.	See Table 1.	See Table 1.	See Table 1.	Plasma corticosterone
Mychasiuk et al. [39]	See Table 1.	See Table 1.	See Table 1.	See Table 1.	See Table 1.	Dnmt1<br>IGF1<br>Pgc1-α<br>Tert
Shin et al. [40]	See Table 1.	See Table 1.	See Table 1.	See Table 1.	See Table 1.	Total-ERK1/2<br>Phosphorolated-ERK1/2

Rafie et al. [42]	Experimental	Male Wistar rats	Weight drop	Animals received either the sham surgery or TBI. The TBI animals were further divided into exercise groups which initiated exercise early (exercised from 1 day post-injury for 4 weeks), late exercise (initiated exercise 1 wk post-injury and exercised for 4 wks), or prophylactically exercised for 4 weeks prior to TBI and continued exercise for 4 weeks after TBI. Motor and behavioural changes were assessed 24 hours after the	Animals exercised 5 days per week for 4 weeks. Each session began at a speed of 2 m/min for 5 min, 5 m/min for the next 5 min and then 8 m/min for 20 minutes, resulting in a 30 min exercise session. Treadmill incline was maintained at 0°.	Serum and CSF quantification of:<br>Malondialdehyde<br>Total antioxidant capacity<br>TNF-α

				last exercise session and animals were sacrificed		
Bharadwaj et al. [43]	See Table 1.	See Table 1.	See Table 1.	See Table 1.	See Table 1.	Prodynorphin<br>CXCL1<br>CXCL2<br><br>Immunohistochemistry of spinal cord tissue for:<br>GFAP<br>IBA-1<br><br>Plasma protein concentrations for:<br>GFAP<br>Ubiquitin carboxyl terminal hydrolase L1 (UCHL1)

mRNA, messenger Ribonucleic Acid; RW, Running Wheel; CA1, Hippocampal Cornu Ammonis 1; CA3, Hippocampal Cornu Ammonis 3; TBI, Traumatic Brian Injury; qPCR, quantitative Polymerase Chain Reaction; MAPKI, Mitogen Activated Protein Kinase 1; MAPKII, Mitogen Activated Protein Kinase 2; ; MAG, Myelin-associated glycoprotein; Nogo-A, Neurite Outgrowth Inhibitor- A; PKA, Protein Kinase A; GAP-43, Growth Associated Protien-43; SYP, Synaptophysin; IGF-1, Insulin Growth Factor-1; Pgc1-α, Peroxisome proliferator-activated receptor-gamma coactivator 1- alpha; Dnmt1, DNA methyltransferase 1; Tert, Telomerase reverse transcriptase; PPCS, Persistent Post-Concussion Symptoms; TNF-α, Tumor Necrosis Factor- Alpha; ; CXCL1/2, C-X-C motif chemokine ligand 1/2; PDYN, Prodynorphin; GFAP, Glial fibrillary acidic protein; ; IBA-1, Ionized calcium binding adaptor molecule 1; UCHL, Ubiquitin C-Terminal Hydrolase L1

Supplemental Table B: Results and conclusions for additional biomarkers not included in the primary tables.

Reference	Relevant Results	Conclusions

Griesbach et al [33]	Exercise following FPI resulted in lower levels of PKC ($p<0.005$), CAMKII ($p<0.0005$), phosphorylated MAPKI ($p<0.005$) and MAPKII ($p<0.005$) compared to the FPI sedentary group.	See Table 1.
Chytrova et al [35]	Exercise normalized myelin associated molecules MAG ($p < 0.001$) and Nogo-A ($p < 0.001$) following FPI compared to the FPI sedentary group. Introduction of a BDNF blockade abolished exercise induced normalization of MAG and Nogo-A.<br><br>GAP-43 and SYP are both downregulated following FPI, however exercise returns the levels of GAP-43 and SYP to the level of sedentary controls (both $p < 0.01$)<br><br>Exercise following FPI normalized hippocampal PKA levels ($p < 0.001$) compared to FPI alone. while blockade of BDNF prevented this upregulation ($p<0.001$).	See Table 1.
Szabo et al [38]	Exercise resulted in decreased accumulation of carbonyl groups compared to a sedentary group following FPI (determined by $p < 0.01$) suggesting decreased oxidative stress.<br><br>Exercise following FPI reduces chymotrypsin like activity of proteasome ($p < 0.05$), 20S alpha content ($p < 0.01$) and Zif268 content ($p < 0.01$) compared to the FIP sedentary group.	See Table 1.
Kim et al [37]	Corticosterone was not altered following TBI with exercise ($p > 0.05$).	See Table 1.

Mychasiuk et al. [39]	Following mild TBI, IGF-1, PGC1-α gene expression in the prefrontal cortex and hippocampus were suppressed, but exercise restored this gene expression to control levels in both male and female animals.<br>Exercise following TBI did not alter DMNT1 expression in either sex in the prefrontal cortex or the hippocampus.<br>TERT expression was supressed following TBI in the prefrontal cortex and hippocampus of the male animals, and remained suppressed with exercise with the exception of within the hippocampus if exercise was initiated immediately following TBI. TERT expression was not supressed following TBI in the female prefrontal cortex, but was lower in the hippocampus. However, hippocampal TERT expression was resolved to baseline with exercise initiated at any time point.	See Table 1.
Shin et al [40]	TBI resulted in a decreased ratio of phosphorylated ERK to total ERK in the hippocampus, while exercise was able to increase all compared to the TBI sedentary animals.	See Table 1.
Rafie et al [42]	Malondialdehyde concentrations in serum and cerebral spinal fluid are increased following TBI ($p < 0.001$), however these concentrations were decreased with exercise initiated 24 hours after injury ($p < 0.001$).<br>Exercise initiated 24 hours after injury also increased serum and cerebral spinal fluid concentrations of total antioxidant capacity ($p < 0.001$) compared to the TBI sedentary group	Exercise initiated early after TBI decreases oxidative stress and pro-inflammatory cytokine TNF-α, which could benefit recovery.

	which saw TBI induced decreases. Additionally, the animals which initiated exercise 24 hours after TBI saw greater increases in total antioxidant capacity compared to animals which initiated exercise 1 week after TBI ($p < 0.01$). TBI-induced elevations of TNF-α were supressed with exercise ($p < 0.001$) with exercise initiated 24 hours after TBI resulting in a greater response than exercise started 1 week post-TBI ($p < 0.05$).	
Bharadwaj et al [43]	Following TBI, lumbar spinal mRNA levels of PDYN, CXCL1 and CXCL2 are increased compared to control animals (all $p < 0.0001$), while exercise treatment decreased the mRNA levels of all biomarkers compared to the TBI sedentary animals (PDYN, $p < 001$; CXCL1, $p < 0.001$; CXCL2, $p < 0.0001$).<br>GFAP staining of the lumbar spinal cords superficial dorsal horns found that exercise following TBI increased the area of GFAP staining compared to the TBI sedentary group ($p < 0.001$), but there was no difference in plasma GFAP.<br><br>There was no difference between any group for area of IBA-1 staining or plasma UCHL levels..	Exercise decreased spinal mRNA expression of CXCL1, CXCL2 and PDYN which have previously been implicated with TBI-induced pain and sensitization of nociceptors.

mRNA, messenger Ribonucleic Acid; RW, Running Wheel; CA1, Hippocampal Cornu Ammonis 1; CA3, Hippocampal Cornu Ammonis 3; FPI, Fluid Percussion Injury; TBI, Traumatic Brian Injury; qPCR, quantitative Polymerase Chain Reaction; CCI, Controlled Cortical Impact; MAPKI, Mitogen Activated Protein Kinase 1; MAPKII, Mitogen Activated Protein Kinase 2; ; MAG, Myelin-associated glycoprotein; Nogo-A, Neurite Outgrowth Inhibitor- A; PKA, Protein Kinase A; GAP-43, Growth Associated Protien-43; SYP, Synaptophysin; IGF-1, Insulin Growth Factor-1; Pgc1-α, Peroxisome proliferator-activated receptor-gamma coactivator 1- alpha; Dnmt1, DNA methyltransferase 1; Tert, Telomerase reverse transcriptase; PPCS, Persistent Post-Concussion Symptoms; TNF-α, Tumor Necrosis Factor- Alpha; ; CXCL1/2, C-X-C motif chemokine ligand 1/2; PDYN, Prodynorphin; GFAP, Glial fibrillary acidic protein; ; IBA-1, Ionized calcium binding adaptor molecule 1; UCHL, Ubiquitin C-Terminal Hydrolase
